# Does Nursing have ‘The Write Stuff’?

**DOI:** 10.1590/1518-8345.0000.3630

**Published:** 2022-07-15

**Authors:** Philip Darbyshire

**Affiliations:** 1 Retired Professor of Nursing, Highbury, SA, Austrália.

Why do nurses write? Acceptable answers are easy, including; sharing knowledge and information, debating professional issues, disseminating research ideas and findings, advancing knowledge and developing professional CVs (*curriculum vitae*). These are perfectly worthy aims but on their own they lead to a professional literature beset by boredom and replete with papers that no-one apart from the authors will ever look at.

Nurses need to write work that people enjoy reading. William Zinsser’s observation from over 40 years ago, in his classic, “On Writing Well” is as true today as it will be in another 100 years: “(The writer) is still stuck with the same old job of saying something that other people will want to read”^1^. This should be both obvious and unarguable, but decades of advice and strictures from academic supervisors, journal editors and manuscript reviewers, that professional writing should be detached, factual, formal, written in third person and referenced to the nth degree have sucked much of the life from nursing’s literature. While researching paediatric palliative care, I vividly remember a paper that noted the: “... remarkable absence of discussion of the importance of parental love in the pediatric intensive care literature”^2^. It was a moment when time almost stood still. How on earth could anyone understand the world of children’s intensive care without understanding the molten, driving, love and fear that grips every parent there? This was the place where parents may see their child alive for the last time. I could almost see the army of writing advisors saying, “Take the word ‘love’ out. It is poorly conceptualised, over-emotional and unsuitable for that scholarly article you are writing.”

It is not that nurses cannot write well. Where nurses write about nursing outside of the confines of the professional literature, the results can be as stunning as they are informative^3^. The tragedy of nursing’s professional literature is that such writing is often seen as “fringe” or “populist” and marginalised as “grey literature”, when in fact it showcases the full colour palette that nurse writers should be using to reveal and explore the countless facets of nursing, care and the profound human experiences of illness, injury, loss, recovery, ageing, nurse-patient/client relationships and so much more.

Professional journals with reams of guidelines and “instructions to authors” are not exclusively at fault here. The situation has worsened exponentially with the growth of predatory publishers and their endless roster of appalling “journals” and “conferences”^4^. There cannot be nurse academic anywhere who does NOT receive the daily email deluge of spam “invitations” from these fraudsters to publish with them, join their “editorial boards” or speak at their “prestigious conferences”. The damage that these fakes wreak is hard to overestimate, not only to nursing’s credibility and self-respect but to the craft of professional writing. Put bluntly, predators will accept anything for “publication” or “presentation”. It matters not one jot how bad the writing is, providing that payment is made. Why would a nurse spend the days, weeks or months required to hone and craft a paper into a coherent, persuasive and articulate piece of work, that is then subject to review, when a predatory journal will publish any first-draft gibberish submitted?

I do not call for unrestrained “purple prose” or a triumph of style over substance, but some style would surely leaven the growing mound of published papers. In other areas of writing, people have their “favourite authors” whose work they anticipate, read and enjoy sharing. These can be journalists, essayists, or novelists but they will certainly have something to say and be able to say it well. Are there many nurse writers whose work is viewed so positively, or whose next paper is so keenly anticipated?

What do we have to look forward to in nursing’s literature? Hundreds of research reports will continue to be published, full of “bloodless findings”^5^ and concluding that “further research is needed in this area”. More earnest opinion pieces will intone that “Nursing leadership is vital”, that “Nursing is at a crossroads”, that “more resources are needed” for something or everything, that poor care is unacceptable or any of a thousand other hackneyed platitudes that we never need to hear again.

I sometimes imagine a nursing literature so full of great writers and writing that nursing could have its own worldwide “Writers Week” or “Book Festival” events attracting not only nurses and health professionals but members of the public who tell us that they have learned so much about nursing, health and care from reading their favourite nurse authors and listening to their podcasts and audiobooks. Does nursing have enough of “The Write Stuff” to ever make this a reality?
